# The polymorphism Val158Met in the *COMT* gene: disrupted dopamine system in fibromyalgia patients?

**DOI:** 10.1097/j.pain.0000000000003313

**Published:** 2024-06-25

**Authors:** Maria Carla Gerra, Cristina Dallabona, Matteo Manfredini, Rocco Giordano, Camilla Capriotti, Alberto González-Villar, Yolanda Triñanes, Lars Arendt-Nielsen, Maria Teresa Carrillo-de-la-Peña

**Affiliations:** a Department of Chemistry, Life Sciences, and Environmental Sustainability, University of Parma, Parma, Italy; b Center for Neuroplasticity and Pain (CNAP), SMI®, Department of Health Science and Technology, Aalborg University, Aalborg, Denmark; c Psychological Neuroscience Lab, Psychology Research Centre, School of Psychology, University of Minho, Braga, Portugal; d Department of Clinical Psychology and Psychobiology, University of Santiago de Compostela, Santiago de Compostela, Spain; e Department of Gastroenterology & Hepatology, Mech-Sense, Clinical Institute, Aalborg University Hospital, Aalborg, Denmark; f Steno Diabetes Center North Denmark, Clinical Institute, Aalborg University Hospital, Aalborg, Denmark

**Keywords:** Val158Met, Fibromyalgia, COMT, Dopamine, Pain intensity, Fibromyalgia comorbidities

## Abstract

The single-nucleotide polymorphism (SNP) *rs4680* in the catechol-O-methyltransferase gene (*COMT*) is a missense variant (Val158Met) associated with altered activity of the COMT enzyme and suggested as a predictive feature for developing some chronic pain conditions. However, there are controversial results on its role in fibromyalgia (FM). Here, the SNP Val158Met was analyzed in 294 FM patients (without comorbidities) and 209 healthy controls (without chronic pain). The concurrent impact of Val158Met genotypes and FM comorbid disorders (depression and sleep impairment) on FM risk were tested. In addition, the genotypic distribution of FM patients in relation to pain intensity was evaluated. The G allele (Val) resulted in being more represented in the FM group (57.8%) compared with the control group (48.8%; *P* = 0.037). Logistic regression highlighted that having the G/G (Val/Val) homozygous genotype was associated with 2 times higher risk of having FM compared with the A/A (Met/Met) carriers (*P* = 0.038), whereas depression and sleep impairment increased FM risk by 12 and 8 times, respectively (*P* < 0.001). However, considering only the FM patient group, the A/A homozygous genotype was significantly associated with severe pain intensity (*P* = 0.007). This study highlighted associations between the SNP Val158Met and both FM and pain intensity, suggesting a link between dopaminergic dysfunction and vulnerability to chronic pain. Further studies should explore this SNP in FM patients in conjunction with COMT enzymatic activity and other symptoms connected with the dopaminergic system such as depression or sleep impairment.

## Abstract 2

The Val158Met polymorphism was significantly associated with fibromyalgia, pain intensity, depression, and sleep impairment. Interconnections between the identified factors and dopamine should be further explored.

## 1. Introduction

Fibromyalgia (FM) is a syndrome characterized by chronic widespread musculoskeletal pain accompanied by additional symptoms, such as fatigue, sleep disturbance, cognitive dysfunction, depression, and the inability to carry out normal daily activities.^5,28^ Affecting 1% to 5% of the population, the disease is more common in women than in men,^13^ with variable age of onset,^15^ possibly with some degree of heritability,^1^ and often other comorbid conditions.^4^ Because hyperalgesia and allodynia are usually found as prominent symptoms,^48^ a neurogenic origin with central manifestations and pain-processing dysfunctions was hypothesized.^8,46^ Peripheral abnormalities may contribute to increased nociceptive activity.^37^ Neuroendocrine and environmental factors and genetic predisposition are likewise suggested contributing to the symptomatology.^9^ Despite the advances in the understanding of the underlying pathological processes, assessment and diagnosis of FM with quantitative biomarkers still represent a gap in the clinical practice,^9^ with as many as 75% of people suffering from this syndrome remaining undiagnosed.^37^

The genetic background has been investigated to explain some of the variance in FM pain and its psychosocial concomitants^2,21^ and patients with FM often show a family history of chronic pain and risk polymorphisms in genes of the nociceptive pathway.^15,22^ Among the genes of FM susceptibility, the catechol-O-methyltransferase (*COMT*) gene encoding the catechol-O-methyltransferase, a key metabolizing enzyme involved in the degradation of catecholamines, has been proposed as a possible predictor of chronic pain development.^10^ It is thought to be involved in central pain processing, mood, and responses to physical and emotional stressors through its direct regulation of dopaminergic pathways, which results in compensatory changes in opioidergic processing in response to pain.^21^ The missense variant Val158Met (*rs4680*) is most commonly analyzed in the *COMT* gene in the context of pain research and was associated with pain symptoms in FM patients.^55^ It causes the substitution of the amino acid valine for methionine at codon 158. It determines 3 possible genotypes with different levels of enzymatic activity^39,44^: homozygous genotype A/A with low activity (Met/Met, L/L), heterozygous A/G with intermediate activity (Val/Met, H/L), and the homozygous high activity G/G (Val/Val, H/H).^23,26^ Hence, the Val/Val genotype gives rise to a higher effective enzyme, clearing catecholamines faster from the system compared with the Met/Met.^44^ The studies performed so far reported conflicting results on the genotype frequencies of this single-nucleotide polymorphism (SNP) in FM.^26,49^ In light of the *COMT*'s crucial role in dopamine metabolism and the link between dopaminergic activity, depression, and sleep problems,^21,22^ the aims of this study were (1) to examine the genotypic distributions and allele frequencies of the *COMT* Val158Met polymorphism in a large sample of patients diagnosed according to the latest FM diagnostic criteria and comprehensively phenotypic characterized, in comparison to unaffected relatives; (2) to assess the potential prediction of FM risk based on *COMT* genotypes and concurrent symptoms associated with dopaminergic activity, such as depression or sleep disturbance; and (3) to evaluate the potential correlation between pain intensity levels in FM patients and the Val158Met genotypes.

## 2. Methods

### 2.1. Sample collection and DNA extraction

Fibromyalgia patients and controls selected for the present research belong to a cohort of 950 Caucasian subjects described in Gerra et al. (2021).^22^ The study was approved by the Ethics Committee of Galicia, Spain (Registration Code: 2013/582), and written informed consent was obtained from all the participants, who agreed to enter the study as volunteers. All the participants were subjected to a phenotype characterization and cases and controls completed a series of questionnaires and were assessed through a systematic clinical interview that included age, height, weight, demographic data, and family history of FM and years since diagnosis. Confirmation of diagnosis in FM patients was obtained following the American College of Rheumatology (ACR) 2016 criteria. Fibromyalgia severity, measures of pain intensity, fatigue, sleep dysfunction, and depression were collected using the Fibromyalgia Survey Questionnaire (FSQ),^52^ The Fibromyalgia Impact Questionnaire (FIQ),^11^ the visual analogue scales (VAS) for pain intensity,^17^ the Pittsburgh Sleep Quality Index (PSQI),^36^ and The Beck Depression Inventory (BDI).^3^

For the present analysis, a group of 503 DNA samples were selected from the full cohort. In particular, 209 healthy controls were selected among the relatives, with no FM diagnosis or presence of other chronic pain conditions, and 294 patients with FM diagnosis, with and without comorbid symptoms/disorders of the syndrome (chronic fatigue, temporomandibular disorders, irritable bowel, depression), but no other concomitant pathologies. Participant age ranged from 18 to 86 years with a mean of 48.1 ± 14.2 years for FM patients and 47.8 ± 14.4 years for controls, although the median for both groups was 49 years. The final FM group was composed entirely of women, mostly siblings; the control group, on the other hand, included 16 men, but not a sufficiently high number to consider gender as a separate category.

Data related to depression (BDI score ≥14 for depressed participants and BDI score <14 for no depressed participants), sleep impairment (PSQI score <15 for absent or mild sleep impairment and PSQI score ≥15 for FM patients with severe sleep impairment), and VAS (VAS score ≥ 6.5 for severe pain intensity and VAS score <6.5 for mild and moderate pain intensity) were also considered. We used categorical variables from these scales given that we were interested in identifying subgroups that may have clinical relevance. In the case of VAS-Pain, the cutoff point was selected according to the recommendation of Boonstra et al.^7^

### 2.2. Genotyping procedure

The polymorphism *rs4680* was genotyped in fibromyalgia patients (n = 294) and controls (n = 209) by real-time polymerase chain reaction (PCR) method. Genomic DNA (gDNA) samples were processed with Taq-Man Genotyping Assays (Thermo Fisher) for the identification of allelic variants. In particular, 6.75 ng of gDNA were amplified in a final volume of 12.5 μL with 6.25 μL of 2X TaqMan Genotyping Master Mix and 0.625 µL of 20X Drug Metabolism Genotyping Assay Mix. Amplification was performed on the QuantStudio3 system (Applied Biosystem, Thermo Fisher Scientific, United States), with the following amplification conditions: 60°C for 30 seconds, followed by 50 cycles at 95°C for 15 minutes and at 60°C for 1 minute and 30 seconds. All genotypes were determined twice and were finally scored based on fluorescence profiles revealing the end point genotyping. Negative controls and reference samples, as positive controls, were included to ensure the accuracy of the analysis. Allelic discrimination was conducted using the algorithm and software supplied by the manufacturer.

### 2.3. Statistical analyses

The χ^2^ test was applied to investigate differences in the genotypic distribution and allele frequencies between FM patients and healthy relatives. The deviations of genotype distribution from the Hardy–Weinberg equilibrium were also assessed. A logistic regression model was then used to evaluate the concurrent impact of *COMT* genotypes, depressive symptoms, and sleep problems on the FM risk. In addition, we assessed, in the sole subgroup of FM patients, the power of SNP *rs4680* genotypes to predict pain intensity levels (severe vs mild-moderate) using a logistic regression model. All the statistical analyses were performed with Stata 15.1 (College Station, TX). Because the 2 groups, FM patients and controls, are women, the sample was not divided into 2 gender subgroups, and thus, all the variables have been evaluated net of gender effect. The threshold for significance was set at *P* < 0.05.

## 3. Results

### 3.1. Genotyping of Val158Met in fibromyalgia patients and controls

Genetic analyses were conducted on 294 FM patients and 209 relatives as controls. Genomic DNA samples collected from these participants were genotyped for the polymorphism *rs4680* (*COMT* gene). Table 1 (A and B) report the results of genotyping. Observing the SNP *rs4680* genotypes distributions, the GG carriers tended more frequently to be associated with the FM group compared with controls (χ^2^ *P* = 0.120) (Table 1A). Statistically significant differences were shown at the allelic level: the G allele is significantly most represented in the FM group (57.8%) compared with controls (48.8%) (χ^2^: *P* = 0.037, Table 1B).

**Table 1:** Genotype distributions (A) and allele frequencies (B) of the single-nucleotide polymorphism *rs4680*, *COMT* gene.

		FM patients	Control subjects	*P* (χ^2^)
		(n = 294)	(n = 209)	
A	Homozygous G/G (ValVal) Homozygous A/A (MetMet) Heterozygous G/A (ValMet)	100 (34%) 54 (18.4%) 140 (47.6%)	57 (24.9%) 52 (27.3%) 100 (47.8%)	0.120
B	Allele G (Val) Allele A (Met)	340 (57.8%) 248 (42.4%)	214 (51.2%) 204 (48.8%)	0.037

FM, fibromyalgia.

The genotype distribution of the Val158Met genotypes detected in the present population was 24.9% for Val/Val, 47.8% for Val/Met, and 27.3% for Met/Met and did not differ significantly from those expected from the Hardy–Weinberg equilibrium (Hardy–Weinberg, χ^2^: *P* > 0.05).

### 3.2. Genetic and environmental impact on fibromyalgia risk

The concurrent impact of depression (BDI score), sleep impairment (PSQI score), and the *COMT* Va158Met genotypes on the FM risk was evaluated by means of logistic regression (Table 2). Age was included as a covariate. The results revealed that the genotype of the *COMT* polymorphism was significantly associated with FM (OR 2.19; *P* = 0.038): in particular, individuals with the G/G genotype (Val/Val) have a 2 times higher risk of having FM compared with those with the A/A genotype (Met/Met). In addition, depression and sleep dysfunction were also significantly associated with FM risk: having depression and severe sleep impairment is associated with 12 times and 8 times higher risk to have FM, respectively. Age also resulted associated with FM risk.

**Table 2:** Logistic regression model testing the simultaneous influence of age, sleep impairment, depression, and *COMT* Val158Met (rs4680) genotypes on the risk of having fibromyalgia.

Logistic regression	No. of obs = 445		
Fibromyalgia	Odds ratio	SE	*P* >|z|
Age	1.043	0.01	<0.001
BDI	12.659	3.559	<0.001
PSQI	7.971	3.049	<0.001
rs4680—*COMT* (ref.cat AA)			
AG	1.509	0.526	0.237
GG	2.198	0.834	0.038
	Wald χ^2^ (5) = 115.9		
	Prob > χ^2^ = 0.0000		

AG, Heterozygous G/A; BDI, Beck Depression Inventory; GG, Homozygous G/G; PSQI, Pittsburgh Sleep Quality Index.

### 3.3. The single-nucleotide polymorphism Val158Met in relation to pain intensity in fibromyalgia patients

The logistic regression using pain intensity levels (severe vs mild-moderate) as dependent variable, for the subgroup of patients with FM, showed that the *rs4680* A/A (Met/Met) genotype was significantly associated with the greater severity of pain, compared with both heterozygous A/G (Met/Val) and homozygous G/G (Val/Val) genotypes (Table 3). Among the FM patients, having G/G or A/G genotypes reduced the risk of developing severe pain by 60% (*P* = 0.040) and 70% (*P* = 0.005), respectively, compared with the A/A (Met/Met genotype).

**Table 3:** Logistic regression model testing the influence of *COMT* Val158Met (*rs4680*) genotypes on the risk to develop severe pain intensity (measured through visual analogue scale).

Logistic regression	No. of obs = 284		
Pain intensity (VAS)	Odds ratio	SE	*P* >|z|
rs4680—*COMT* (ref.cat AA)			
AG	0.306	0.130	0.005
GG	0.402	0.178	0.040
	Wald χ^2^ (2) = 7.81		
	Prob > χ^2^ = 0.0201		

AG, Heterozygous G/A; GG, Homozygous G/G; VAS, visual analogue scale.

## 4. Discussion

This study showed that the SNP Val158Met was present more frequently in the FM patients compared with their relatives, and it was associated with the pain intensity. Furthermore, the G/G (Val/Val) homozygous genotype was associated with a 12-time increased risk for depression and an 8-time increased risk for sleep disturbance, suggesting a link between dopaminergic dysfunction and the vulnerability to develop FM. The G allele (Val/Met or Val/Val genotypes) seemed to cause reduced risk for developing severe pain, compared with the A/A (Met/Met) genotype.

### 4.1. Previous findings on the Val158Met single-nucleotide polymorphism of the *COMT* gene

The *rs4680* SNP of the *COMT* gene has been widely studied in connection with multiple pain diseases and pain measures. Previous results considering the *COMT* gene variants in FM patients provided significant heterogeneity. The majority of the studies in FM reported inconsistent evidence or no differences in the genotypic distributions.^14,18,20,24,38,49^ A systematic review and meta-analyses could not support an association of the *COMT* gene Val158Met polymorphism with FM risk.^54^ Conflicting results also emerged: some studies reported the presence of the A allele (Met) significantly more frequent in patients than controls^23,26,41^ and associations of the A/A (Met/Met) genotype with higher FIQ scores in FM patients compared with controls.^2^ Despite these previous results, it should be noted that the available data on *COMT rs4680* and FM were heterogeneous because of several discrepancies. Although FM was accepted as a disease in 1987^53^ with specific diagnostic criteria,^51,52^ patients included in studies may have additional concomitant pathologies, the sample size was usually too small, and patients' characterization was often incomplete.^26,49^ In addition, heterogeneous populations have been included, showing different allele frequencies depending on the ethnic group.^25^ No solid evidence is therefore available on the Val158Met SNP.

### 4.2. Potential implications of the Val158Met single-nucleotide polymorphism on the dopaminergic pathway

To our best knowledge, this study is the largest cohort study of well-characterized FM patients where the *COMT* gene polymorphism Val158Met is analyzed. The G allele of the Val158Met SNP, corresponding to the valine in the amino acid sequence and the high-activity enzyme, resulted most represented in the FM group compared with the control group of relatives without FM. The catechol-O-methyltransferase enzyme is responsible for the degradation of catecholamines and the Val/Val genotype entails breaking down dopamine 40% faster than the Met/Met genotype^12,35^; consistently, *COMT* activity in the human dorsolateral prefrontal cortex was higher in G (Val) allele carriers, compared with (A) Met allele carriers.^42^ The higher enzymatic activity in G (Val) allele carriers implies a shorter dopamine availability at the synapses.^6^ How dopaminergic pathways are involved in pain modulation has been recently and extensively described.^33^ Dopamine could be available shorter in FM patients with the G/Val allele than in the case of the A/Met allele: this shorter availability of neurotransmitter at the synapse (caused by faster degradation performed by the *COMT* enzyme) can largely influence neuronal activity and thus could affect descending pain pathway. The fact that the perception of nociceptive information and the affective symptoms of chronic pain were modulated by the mesolimbic dopaminergic system was already proposed.^45^

The higher frequency of G/G (Val/Val) carriers in FM groups compared with controls was also confirmed in the logistic regression model, in which also depression and sleep impairment symptoms showed significant associated with the disease risk. Interestingly, dopamine system dysfunction has been associated with both depression symptoms^30^ and sleep problems.^16^ In addition, recent analysis showed an effect of the G/G genotype and severe stressful life events on the severity of depression in the first episodes of psychosis.^47^ As previously evidenced, the simultaneous evaluation of environmental and genetic factors highlighted depressive symptoms and sleep disorders as factors contributing to FM risk in conjunction with the genetic effect.^22^

### 4.3. Val158Met single-nucleotide polymorphism genotypes relate to pain intensity in fibromyalgia

Interestingly, considering only the FM patients, the homozygous A/A (Met/Met) carriers were characterized mostly by severe pain, whereas the G/G (Val/Val) and A/G (Met/Val) carriers had mostly mild and moderate pain intensity. This result seems consistent with previous research investigating this SNP in association with pain intensity. The recent review and meta-analysis conducted by Vetterlein and coworkers reported exactly that analyzing separately chronic pain patients, A (Met) allele homozygous patients were significantly more pain sensitive compared with heterozygotes.^50^ The association of Met/Met individuals with increased pain sensitivity was also confirmed by Jensen and colleagues, who showed Met/Met carriers reporting significantly more pain compared with Val/Val carriers after thermal pain stimulation.^27^ The low *COMT* activity genotype (Met/Met) was also reportedly associated with lower pain thresholds compared with heterozygotes,^50^ with a more severe form of the disease in the Spanish population^29^ and exhibited significantly higher levels of self-reported fatigue.^20^ Even in fMRI studies, painful stimuli applications demonstrated stronger activation of brain areas critical in pain processing and descending pain modulation in Met/Met carriers.^34,43^

This result suggests that FM patients may be characterized by a more complex pain processing system compared with other pain patients, with interconnections between the dopamine system and pain pathways.

It is noteworthy that animal studies demonstrated dopamine D3/D2 receptor-preferring agonists to diminish both pain and mood-related dysfunctions associated with FM.^40^ However, no sufficient evidence is still available in humans: only a mindfulness-based psychological intervention was applied with the purpose of restoring low dopamine in FM patients.^31^

### 4.4. Limitations

One limitation of this study was that no data on other SNPs within the *COMT* gene were available. This has prevented to perform haplotype analyses, which would allow studying composite effects from combinations of multiple SNPs along the gene. Studies, in fact, reported specific haplotypes, including the *rs8046* SNP, significantly associated with FIQ score^49^ or with pain sensitivity.^19,32^ Another limitation is that the controls used were healthy relatives, but not necessarily sibling pairs, thus preventing the identification of matched controls for each patient. Furthermore, the fact that the study was conducted only with women, to ensure a more homogenous sample, limits its generalizability to male FM patients. Finally, it was not possible to conduct an epigenetic analysis of the *COMT* gene sequence; however, especially in complex diseases such as FM, epigenetic factors can be real mediators of environmental exposure and provide insights into the underlying disease mechanisms.

## 5. Conclusion and perspectives

**Figure 1.**
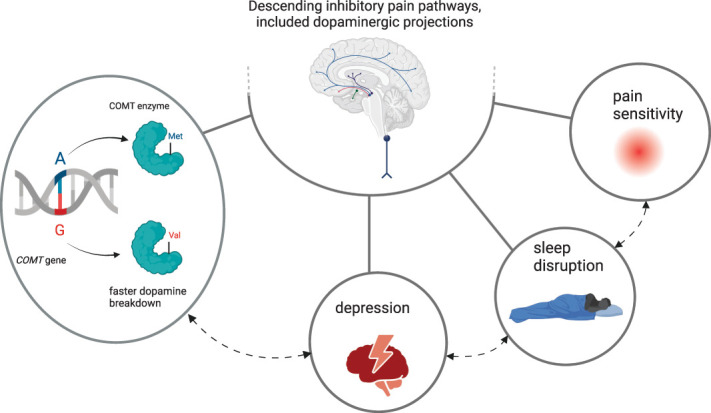
The hypothesis of multiple factors influencing the top–down pain modulation in FM disease, including the *COMT* SNP Val158Met, pain sensitivity, depression, and sleep disruption. *COMT*, catechol-O-methyltransferase; FM, fibromyalgia; SNP, single-nucleotide polymorphism.

A higher frequency of the G (Val) allele was found in FM patients as compared with controls, the A/A (Met/Met) genotype was associated with severe pain intensity, and the Met/Val and Val/Val genotypes were associated with mild-to-moderate pain intensity. Furthermore, the G/G (Val/Val) homozygous genotype was associated with a 12-time increased risk for depression and 8-time increased risk for sleep disturbance. This could suggest links between dopaminergic dysfunction and FM symptoms (Fig. 1). Diagnosis, understanding of pathophysiological pathways, and treatment of FM have been hampered by the lack of biomarkers. Thus, these results are particularly significant, providing valuable insights into the genetic contribution to FM, using a large sample and a complete phenotyping of the participants. Besides, given the heterogeneity of FM patients, the identification of genetic profiles, in association with clinical profiles, helps to stratify the patients and represents a step forward toward personalized medicine. Taking into account alleles and genotypes specifically associated with FM could allow for the definition of more effective and tailored therapies for each patient.

## Conflict of interest statement

The authors have no conflicts of interest to declare.
